# Using a Person‐Centred Approach to Adapt a Digital Therapy for Inflammatory Bowel Disease (COMPASS‐IBD)

**DOI:** 10.1111/hex.70818

**Published:** 2026-08-21

**Authors:** Sophie Harding, Harinder Singh, Katrin Hulme, Natasha Seaton, Abigail Wroe, Alexa Duff, Joanna Hudson, Rona Moss‐Morris, Annie S. K. Jones

**Affiliations:** ^1^ Health Psychology Section, Department of Psychology, Institute of Psychiatry, Psychology and Neuroscience King's College London London UK; ^2^ Lead Patient and Public Involvement Representative King's College London London UK; ^3^ Psychological Medicine Unit, St Mark's National Bowel Hospital London North West University Healthcare NHS trust London UK; ^4^ Faculty of Medical and Health Sciences, Psychological Medicine University of Auckland Auckland New Zealand

**Keywords:** anxiety, cognitive behavioural therapy, depression, digital health, inflammatory bowel diseases, patient participation

## Abstract

**Background:**

Patient and public involvement (PPI) enhances the relevance, acceptability and implementation potential of interventions.

**Objective:**

To describe the adaptation of a cognitive‐behavioural therapy (CBT) based intervention (COMPASS) into an inflammatory bowel disease (IBD)‐specific version (COMPASS‐IBD), in partnership with individuals with IBD and healthcare professionals (HCPs).

**Setting and Participants:**

Twelve individuals with IBD, recruited through previous COMPASS PPI networks and wider outreach to the IBD community, and three HCPs contributed to an iterative adaptation process.

**Design:**

A four‐stage iterative adaptation process was conducted. First, an online workshop, one‐to‐one calls and clinic observations were conducted to explore the specific challenges of living with IBD and insights from these discussions were then used to guide adaptations. Proposed changes were reviewed through email and phone correspondence with PPI to check for acceptability and suitability. Finally, a post‐process reflection with collaborators was undertaken to inform future work.

**Results:**

Key themes included the invisible and unpredictable nature of IBD, symptom burden, stigma and isolation. These informed IBD‐specific patient stories and refinement of content to enhance relevance and specificity. Sixty‐four recommendations were made, of which 56 were implemented.

**Conclusions:**

Collaboration with PPI and HCP partners identified IBD‐specific needs and ensured that COMPASS‐IBD was acceptable and contextually appropriate. Embedding lived experience within intervention adaption may help to enhance implementation and engagement for interventions supporting individuals living with long‐term health conditions.

**Lived Experience or Public Contribution:**

Our PPI collaborators were involved throughout the study, via an online workshop, one‐to‐one calls, and follow up email correspondence. They identified key challenges of living with IBD, reviewed adaptations and provided iterative feedback on changes to COMPASS‐IBD. Their input shaped the patient stories, programme content, and the framing of IBD‐specific issues, ensuring the relevance, tone and acceptability of the adapted intervention. PPI contributors also supported with the interpretation of findings and refinement of the manuscript. This work documents a meaningful partnership in the adaptation of a digital intervention.

**Clinical Trials Registration:** The wider study was registered on ClinicalTrials.gov (NCT05330299).

## 1 Introduction

Inflammatory Bowel Diseases (IBD), including Crohn's disease and ulcerative colitis, are chronic inflammatory conditions of the gastrointestinal tract [1], affecting over 600,000 people in the UK, with this number continuing to rise [2, 3]. Symptoms, including chronic diarrhoea, abdominal pain, rectal bleeding and fatigue [4], can be unpredictable, invisible, and often stigmatised [5]. There is currently no cure for IBD, and these lifelong conditions typically present with a relapse and remission cycle [6], with periods of heightened symptoms referred to as a ‘flare‐up’. Living with IBD can therefore be very challenging and have a big impact on individual's daily lives, relationships and activities [7]. The wide‐ranging impact of IBD can also have a significant impact on mood and wellbeing [8]. Prevalence of depression (32.1%) and anxiety (25.2%) in people living with IBD (pwIBD) is significantly higher than in the general population [9].

The presence of co‐morbid mental health conditions in pwIBD has far reaching impacts, with links to increased usage of healthcare, increased steroidal use [10], risk of relapse into active disease [10] and absence from work [11]. Despite the impact of psychological distress in pwIBD at a societal, healthcare, and individual level, the burden of physical IBD symptoms can mean that psychological wellbeing is overlooked within IBD care [12]. This need for holistic integrated care to support psychological wellbeing in IBD is acknowledged by clinicians [13, 14] and has been highlighted as a core target for UK IBD care standards [15]. Despite this recognition, barriers such as administrative load, time‐restriction, lack of understanding and training on providing psychosocial support [13], and the normalisation of depression in the IBD population [16] can prevent the delivery of holistic care. A report by IBD UK exploring the provision of support with over 10,000 patients and 166 IBD services, identified that only one in ten patients reported being asked about their emotional coping during their care, and just 2% of IBD services reported having adequate psychologists in post to meet the recommended standards [17].

Digital interventions provide a scalable way to increase access to psychological support for people living with IBD, with the ability to reduce time (for both patient and therapist) and travel costs (a potentially significant barrier for pwIBD [18]) whilst still providing effective support. Research also indicates that therapist‐guided digital interventions for mental health support can be both effective and comparable to face‐face cognitive behavioural therapy (CBT) [19].

COMPASS is an online, self‐guided 12‐week CBT‐based digital intervention with therapist support (six 30‐min sessions), that has been designed to treat psychological distress (symptoms of depression and anxiety) related to living with long‐term health conditions (LTCs) [20]. COMPASS is based on the transdiagnostic model of adjustment to LTCs (TMA‐LTC) [21], which recognises that distress in the context of an LTC may be driven by unique and chronic stressors, such as the ongoing management of symptoms [22, 23]. Accordingly, COMPASS uses CBT‐methods to target these specific stressors to support patients experiencing distress in the context of living with an LTC [20]. A full outline of the intervention is reported elsewhere [20], briefly, it consists of 11 modules, divided into four quadrants (North, East, South, West) with sessions using psychoeducation, patient stories and goal setting based on CBT principles [20], outlined in Table 1.

**Table 1:** A brief overview of COMPASS modules, therapeutic targets and examples of cognitive‐behavioural therapy (CBT) techniques and interactive tasks used.

Module	Target	Examples of CBT techniques used	Examples of interactive tasks
Navigating compass	Introduction to CBT processes associated with distress to identify ones relevant to user	Assessment Formulation Guided discovery	Personal five area model Problem solving Goal setting
North—Navigating change and uncertainty			
Managing uncertainty	Uncertainty around symptoms and health outcomes	Problem solving Emotion‐focused coping	Problem solving
Power of thoughts	Unhelpful/inaccurate beliefs around health condition	Cognitive restructuring	Identifying thoughts Challenging thoughts
East—Even keel			
Achieving routine	‘Boom or bust’ patterns of activity	Self‐monitoring	Activity diary
Managing symptoms	Symptom focusing	Attentional techniques Relaxation	Personal model around pain
Emotions	Normalising distress and acknowledgement of emotions	Behavioural activation Acceptance	Feel good model Personal emotional model
South—Support			
Strengthening personal relationships	Social support from others	Assertive communication	Support network
Making use of professional support and information	Relationship with healthcare team Helplessness around illness management	Assertive communication Care coordination	Increasing assertiveness
West—living well			
Healthy lifestyle	Positive health behaviours	Key health behaviours	
I am me, not my LTC	Self‐compassion Self‐efficacy Self‐esteem	Cognitive restructuring Motivational interviewing	Self‐compassion task Problem solving
Managing stress	Normalising stress Helpful coping strategies	Relaxation Self‐management skills	Relaxation exercises

In a randomised control trial (RCT) where COMPASS was delivered to patients with LTCs (IBD, psoriasis, chronic kidney disease and multiple sclerosis), there was significant demand for support from the IBD population who made up 49.5% of the sample (*n* = 96), and who were recruited in just 2 days [24]. The study found that patients who received COMPASS, including people with IBD, had moderate treatment effects (standardized mean difference of 0.71, 95% CI 0.48–0.95) on the primary outcome of psychological distress [24].

The demand, lack of provision, and the promising findings from previous COMPASS studies with the IBD population [20, 22, 24] suggested tailoring a version of the COMPASS programme for the IBD population (COMPASS in IBD; COMPASS‐IBD) could further improve its effectiveness, relevance and accessibility as psychological treatment for pwIBD. COMPASS‐IBD was subsequently utilised in a novel integrated treatment pathway, tested in an implementation study in a gastroenterology clinic in the UK, the outcomes of which are reported separately [25].

This study reports the process used to adapt the original transdiagnostic COMPASS intervention into COMPASS‐IBD, an IBD‐specific version targeted at supporting pwIBD experiencing illness‐related distress. A co‐design approach was employed, working alongside Patient and Public Involvement (PPI) members and healthcare professionals (HCPs) to centre the experiences of pwIBD in the collaborative adaptation process. This approach was grounded in the Person Based Approached (PBA) [26], particularly the process of ‘intervention optimisation’ that emphasises the importance of collaborating with the target population and key stakeholders [27, 28] in the tailoring of interventions to understand needs and wider social context, increase acceptability and effectiveness [29] and minimise barriers to engagement [30]. This approach also follows UK Medical Research Council (MRC) guidance on the development of complex interventions, which also prioritises the inclusion of stakeholders as collaborators to ensure the relevancy of interventions in real‐world settings [31].

The importance of reporting on intervention adaptation processes, in collaboration with PPI and stakeholders, is threefold. Firstly, there is a scarcity of research reporting on the design and implementation of digital health interventions, despite the recognition for their need to support advancements and share learnings in this space [18]. Second, while meaningful involvement of people with lived experience is increasingly common in intervention development, there remains somewhat limited systematic document of how their feedback translates to specific, practical adaptations [29]. Third, tailoring pre‐existing interventions for new populations can efficiently utilise developed resources (such as a digital intervention like COMPASS) with limited costs and resource. This manuscript aims to provide a transparent description of the adaptation process used to create COMPASS‐IBD, including details of how this was informed by collaboration with PPI and key stakeholders. This worked example may guide other researchers in utilising co‐design approaches to adapting digital health interventions.

## 2 Materials and Methods

**Figure 1:**
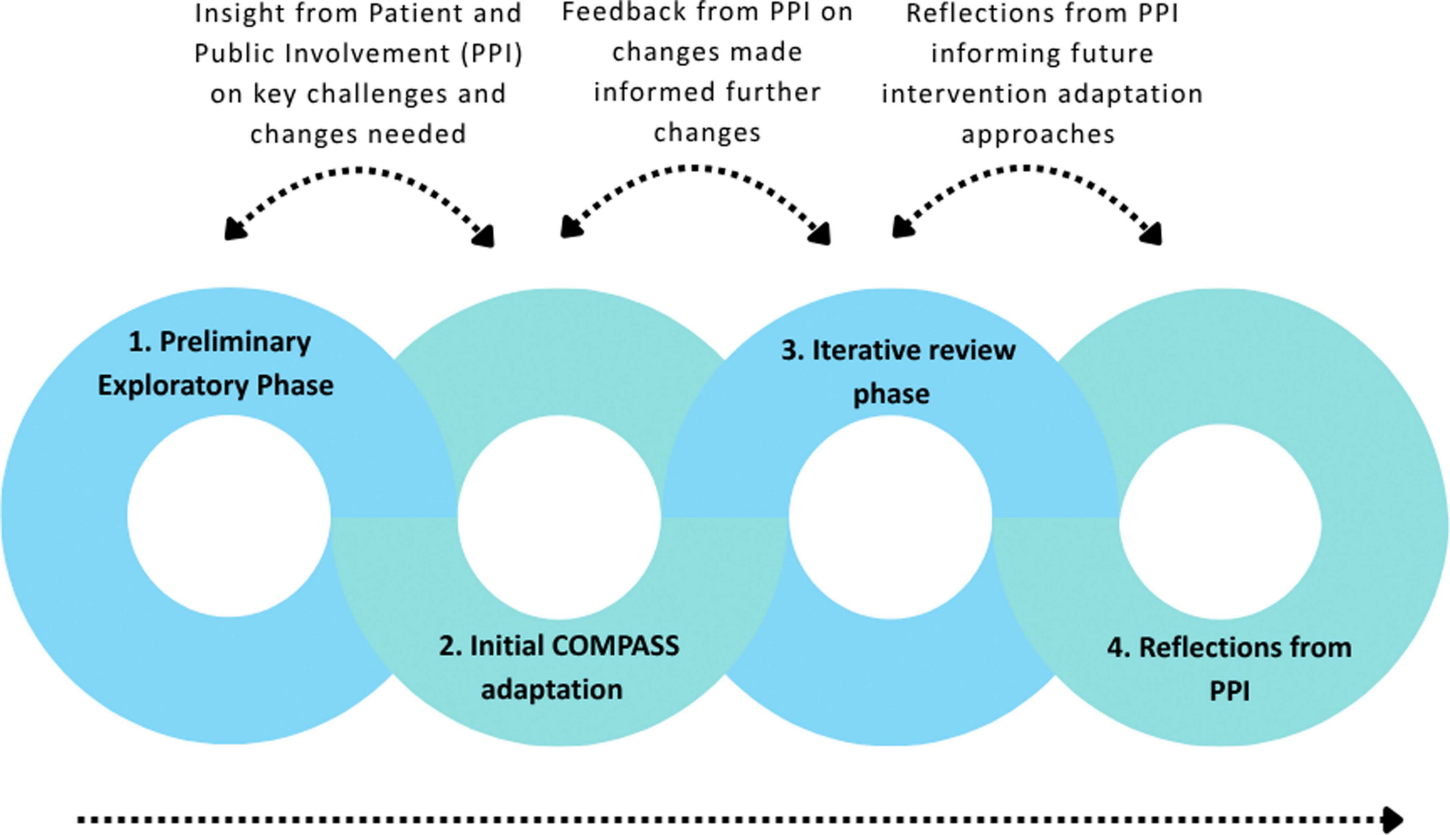
The adaptation process of COMPASS IBD and collaboration with patient and public involvement (PPI) members.

A person‐centred co‐design approach [28, 32] was employed in the study, drawing from the key stages of the MRC framework of development, feasibility/piloting, evaluation, and implementation [31] to structure a study‐specific four‐phase process centred on transparent documentation of intervention adaptation process. This included: (1) the preliminary exploratory phase, developing a greater understanding of the patient experience and psychosocial challenges for pwIBD, (2) the initial COMPASS adaptation phase, where these insights informed adaptations to an IBD‐specific version of the COMPASS programme, including the creation of tailored content alongside PPI members and HCPs, (3) an iterative review phase, where adaptations were reviewed for acceptability and suitability [27, 33] and (4) a post‐process reflection with pwIBD to inform future collaboration. People with IBD and HCPs were collaborators at each stage, as shown in Figure 1. The core research team was made up of health psychology researchers and clinical psychologists working within NHS Gastroenterology services. The Guidance for Reporting Involvement of Patients and Public 2—Short Form (GRIPP2) [34] checklist has been used to systematically report the role and involvement of PPI members in this work (Supporting Information S1: Table S1) and PBA [26] methods were embedded across all phases to ensure iterative, user‐centred optimisation while retaining COMPASS's core therapeutic functions.

This work was part of a wider implementation study conducted between December 2022—July 2024 that received ethical approval from the Health Research Authority and Health and Care Research Wales (HCRW) (IRAS312818, REC reference22/NW/0224). All PPI members were treated as collaborators in the research process rather than research participants and were compensated for their contributions in accordance with NIHR guidance [35]. As co‐developers of the intervention, PPI members were informed that their contributions would be a part of research outputs, with all quotes paraphrased and survey feedback summarised to maintain anonymity.

### 2.1 COMPASS‐IBD Advisory Group

The COMPASS‐IBD advisory group included twelve PPI members living with IBD (three men and nine women; see Table 2 for overview) and key stakeholders included three HCPs (two dieticians and a clinical fellow pharmacist) from the outpatient gastroenterology service where the wider implementation study was based [25]. Of the PPI group, ten had previously completed COMPASS therapy as part of other research projects with the research team [20, 22] including one member who acted as PPI Lead and Co‐Investigator for the wider project and implementation study. Two additional members were identified through the PPI Lead's IBD networks and had no prior COMPASS exposure. This paper focuses specifically on how collaboration with the PPI group and HCP insights informed the adaptation of COMPASS into an IBD‐specific version (COMPASS‐IBD).

**Table 2:** Characteristics of patient and public involvement (PPI) members (*n* = 12).

Characteristics	
Sex, *n* (%)	
Female	9 (75%)
Male	3 (25%)
Age (years), mean (SD)*	44.3 (10.5)
Ethnicity, *n (%)*	
White British	9 (75%)
White Other	1 (8.3%)
Asian/Asian British	2 (16.6%)
IBD Type, *n* (%)	
Crohn's Disease	7
Ulcerative Colitis	5
Years living with IBD, mean (SD)*	17.7 (12.74)

* Missing data (*n* = 2).

### 2.2 Adaptation Process

#### 2.2.1 Phase 1—Preliminary Exploratory Phase

The initial exploratory phase aimed to develop an in‐depth understanding of living with IBD and to identify the most relevant psychosocial challenges for pwIBD. This phase drew on multiple methods, including a workshop and individual interviews with PPI members, field observations of outpatient consultations and drawing from previous qualitative evidence from pwIBD who used COMPASS [36].

##### 2.2.1.1 Workshop

The 2‐h online workshop conducted via Microsoft Teams involved five PPI members and five facilitators (three senior qualitative researchers and two junior researchers). Discussions were structured around core COMPASS content. This included exploring perspectives on: (i) Navigating change and uncertainty in IBD (ii) Working towards balance (focused on emotions towards and management of symptoms in IBD) (iii) Making use of support (focused on how to identify, access and effectively use support available), and (iv) Striving towards general wellbeing and good self‐management (including a focus on self‐confidence and self‐compassion) [22].

All PPI workshop attendees had prior COMPASS experience, therefore discussions focused on what they found most and least helpful in the original intervention, what content could be made more IBD‐specific, and any potential new content areas or elements (such as modifying patient stories and focused goal setting). After an initial full group discussion, attendees were divided into three ‘breakout’ rooms with equal numbers of PPI group members and facilitators. Each breakout room focused on specific sections of COMPASS (North/East/South/West) to ensure breadth of content covered and to allow facilitators to capture perspectives from all attendees. In a final whole group discussion, the facilitators summarised key points from each breakout room discussion and asked for any further feedback around key challenges of living with IBD, or comments from the wider group. The session ended by the group agreeing on a set of priorities regarding psychosocial challenges in IBD to guide intervention tailoring.

##### 2.2.1.2 Interviews

To supplement the workshop findings, one‐to‐one telephone interviews were conducted with three further PPI members (ranging from 30–60 min) between March–April 2022. The interview guides (Supporting Information S1: Table S2) followed the same structure as the workshop, exploring individual experiences and challenges of living with IBD mapped to core COMPASS content. Two of the three PPI members interviewed had no prior knowledge of COMPASS, to include a more diverse perspective.

##### 2.2.1.3 Further Exploratory Work

Field observations of patient consultations (six 1–3‐h clinics) were also conducted by members of the research team (S.H. & N.S.) from March to April 2022, who shadowed HCPs (dieticians, pharmacists, clinical nurse specialists and psychologists) within the outpatient gastroenterology service where the implementation study was to be based. In addition, existing qualitative interviews from previous COMPASS [36] work, with individuals who had used the transdiagnostic version, were also consulted to supplement data collected in the observations, the workshop and interviews.

Detailed field notes from this phase were recorded by researchers, with a focus on highlighting key challenges, experiences and insights raised by PPI and HCPs. All findings were collated by SH, then reviewed by the research team to ensure all key concepts, points, and discussions that arose during the exploratory phase had been collected and recorded appropriately.

#### 2.2.2 Phase 2—Initial COMPASS Content Adaptation Phase

Key findings from Phase 1 were mapped on to the existing topics of the COMPASS programme to highlight potential areas for adaptation (Table 3). Suggested tailoring ranged from simple text edits (e.g., to reference IBD instead of LTC) to the creation of new content within COMPASS modules (see Results section and Table 4 for full details of adaptations made). Core CBT therapeutic strategies were retained and modifications focused on developing IBD‐specific contextual content and examples to sit within the existing framework. These initial suggestions for tailoring were discussed with the wider research team, who agreed on priorities for adapting content before the lead researcher (SH) edited intervention sections to incorporate adaptations. To complete this, content from the original COMPASS site was uploaded to Microsoft Word, to facilitate easily accessible editing and reviewing for the research team and wider advisory group.

**Table 3:** Phase 1 Key findings: overarching challenges and illustrative quotes paraphrased from patient and public involvement (PPI) members.

Key challenges	Examples and paraphrased quotations from PPI members
Fear of having an accident	**“** *Having an accident is a primary concern*.**”** Planning is a big aspect of living with IBD (i.e., the need to plan every day before leaving the house where incontinence is experienced). For example, travel can be particularly difficult because of needing to be near to a toilet.
Guilt around having a long‐term illness	Common feelings of guilt related to living with a long‐term condition, particularly fatigue, and being unable to participate fully in daily life, especially in parenting or partner roles.
Impact of IBD on daily life and work	Frustration at not being able to live life as you desire to because of feeling unwell with IBD. It can be easy to slip into cycles of feeling overworked and fatigued.
Stigma and embarrassment	Embarrassment and shame around symptoms are common. Important for pwIBD to accept IBD and address feelings of stigma. Body image can become severely impacted ‐ pwIBD describe feeling ‘*disgust*’ toward self.
Societal misunderstanding of IBD	Common difficulties around the lack of understanding of the chronic nature of IBD – e.g. **“** *Comments like ‘get well soon’ can be unhelpful*.”. It can be frustrating to keep explaining what IBD is.
A lack of control over IBD	The unpredictability of IBD (specifically fluctuating symptoms) was described as one of the most difficult challenges to cope with—‘*I feel like I've spent my life with no control*.’ Unpredictability makes consistency in daily life difficult. PwIBD often spoke of trying to **“** *control the controllable* **”** and needing to accept that some aspects of IBD remain outside of their control.
Difficulty in disclosing or discussing IBD	Discussing IBD can be difficult and embarrassing. Workplace conversations could be particularly challenging, with perceptions that employers felt uncomfortable with conversations about IBD. Conversations with healthcare teams can be difficult, whereby pwIBD may not always feel supported regarding the challenges they face.
Worries about the need for future surgery/procedures	PwIBD express ongoing worries about their future care and health, including the need for surgeries/procedures. **‘** *Spiralling* **’** when experiencing symptoms, worrying that these signal declining health or a flare up.
Understanding IBD and your body is important	PwIBD discussed the need to accept bodily changes when living with IBD, to prevent feelings of guilt or remorse and the ‘*need to learn to listen to body*’ to best manage your IBD.
Social burden/disruption	PwIBD can feel isolated by the impact of their illness, particularly given others do not understand their symptoms and experiences. “*It can feel like always missing out*.”
Sex and relationships	*“The idea of dating again would be very daunting*.” Sharing experiences of IBD was described as both positive and negative: while disclosure can enable social support, it may also evoke shame, worry and embarrassment.
Difficulty adjusting to diagnosis	Initial diagnosis can be a difficult period, associated with feeling psychologically ‘*low*’ and overwhelmed, this can last for years. Coming to terms with life after diagnosis was described as challenging—‘*Normal feels far away*.’
IBD and mental health	The continued impact of IBD on all aspects of life can feel all encompassing. IBD can take the enjoyment out of daily life. Activities once enjoyed are now linked to feeling unwell or being in a flare.

#### 2.2.3 Phase 3—Iterative Review Phase

Initial drafts of content changes for each COMPASS intervention section were reviewed by both PPI (*n* = 9) and HCP (*n* = 3) members of the advisory group. To increase inclusivity and accessibility for all advisory group members, feedback could be provided in several ways: (1) directly reviewing and suggesting changes in the Microsoft Word documents, (2) comments via email, or (3) via video/phone call discussion. Advisory group members were explicitly asked if adaptations felt relevant, reflective of their experience (as a person living with IBD or HCP working in IBD) and if the content was appropriate, accessible and easy to digest. Advisory group members were encouraged to change sections if they felt it was needed, and to provide some detail into why they felt the change was needed. Each section was sent out to be reviewed by at least two members of the advisory group. This phase was iterative; revisions were made and re‐circulated to members to ensure these reflected their feedback.

#### 2.2.4 Phase 4—Feedback on Advisory Group Process From PPI Members

The researchers also aimed to acknowledge and explore the perspectives of participating in a collaborative adaptation process among PPI group members. After the adaptation process was complete, PPI members were invited to complete a short survey via the online survey platform Qualtrics. The survey asked about the quality of communication from the research team, clarity of their role in the process, any positive and negative aspects they felt about their role and their perceived value of their role (including feedback on compensation received). Response formats were either free‐text or used 4‐point scales (‘Not at all’, ‘Somewhat’, ‘Quite’, ‘Very’; full survey can be found in Supporting Information S1: Table S3).

## 3 Results

### 3.1 Phase 1—Preliminary Exploratory Phase

#### 3.1.1 Phase 1 Key Findings

Findings from Phase 1 were collated and summarised into thirteen overarching key challenges for pwIBD (Table 3). Key challenges highlighted included the societal misunderstanding of IBD (including by loved ones), which can manifest as unhelpful comments to pwIBD such as ‘get well soon’. The PPI group described these comments as resulting in feelings of frustration and weariness given the relapsing nature of IBD. The importance of getting to know your body when living with IBD was also highlighted, whereby PPI members highlighted that increased knowledge leads to greater patient empowerment. Data also emphasised that acknowledging, accepting and understanding the lifestyle adjustments required after an IBD diagnosis, was a process which can be difficult and take time. The impact of IBD on social issues was also apparent, including the disruption in daily activities, work and the impact IBD can have on sex, relationships and dating.

### 3.2 Phase 2—Initial COMPASS Content Adaptation Phase

The key challenges, concepts and specific examples highlighted in Phase 1 were used to inform adaptations the COMPASS programme, alongside the common challenges highlighted in clinical observations.

References of ‘LTC’ or ‘illness’ were changed to ‘IBD’ throughout the programme. More substantive content adaptations included tailoring patient stories, to reflect IBD‐specific symptoms, challenges, perspectives and behaviours highlighted in Phase 1. For example, the impact of steroid medications on sleep and weight fluctuation were incorporated in a patient story (see Table 4), both to acknowledge the difficulties associated with IBD medication side‐effects and to offer practical tips on how to manage these. Core CBT therapeutic components in each section were preserved but were modified to provide IBD‐specific scenarios. For example, in cognitive restructuring exercises in ‘North’ the case study of catastrophic thoughts focused on worries around a colonoscopy procedure. Content considered less relevant in the original COMPASS version, such as the ‘Graded Exercise’ section, were removed and replaced with content addressing more IBD‐specific symptom management. In addition, new resources were created to address issues raised by PPI group members, such as how to manage unhelpful comments from people about IBD. Examples of how key challenges identified in Phase 1 were mapped onto COMPASS sections and resulting adaptations are shown in Table 4.

**Table 4:** Summary of Phase 2 adaptations made to original COMPASS content.

Key challenge	Subthemes	Section changed	Example adaptation
1. Fear of having an accident	Having an accident in public; Creating a plan	South‐ Strengthening personal relationships	*‘Adrian's story’ edited to focus on worries about sex after an episode of incontinence*.
2. Guilt around having a long‐term illness	Impact of IBD on relationships with family and friends; Guilt regarding limitations with work performance or attendance	West—I'm me not my LTC	*‘Sami's story’ edited to include example thoughts, reflecting feelings of failure and being a burden*
		Navigating COMPASS	*‘Leila's story’ expanded to explore the cyclical nature of IBD through an ‘all or nothing’ pattern of guilt and overworking on ‘better’ days*.
3. Impact of IBD on daily life and work	Feeling overwhelmed; Taking medication; Dietary impact; Exercise; Fatigue	North— Managing Uncertainty	*‘Ali's story’ edited to focus on feelings of uncertainty and a want for control over body when experiencing symptoms, showing how to adjust and manage anxieties*.
		Navigating COMPASS	*Included specific examples of medications, such as steroids, and their effects on sleep and weight throughout*.
		South— Managing professional relationships	*‘Luke's story’ edited to discuss the impact of fatigue and ‘brain fog’ during appointments, illustrating how to advocate for oneself*.
4. Stigma and embarrassment	Stigma/taboo, Embarrassment/shame	South – managing professional support	*‘Luke's story’ edited to provide information on how to address uncomfortable topics with a nurse*.
5. Societal misunderstanding of what IBD is	Lack of understanding of IBD (e.g. ‘Get better soon’ comments)	South— Strengthening personal relationships	*‘Riley's story’ edited to address common misunderstanding around IBD and the isolating nature of these experiences, with an emphasis on importance of communication*.
		South— Strengthening personal relationships	*‘Kaden's story’ edited to highlight the experience of appearing ‘well’ to others whilst managing invisible IBD symptoms*.
		New resource— Managing unhelpful comments	*Developed to help manage unhelpful comments, including examples, validation of emotional responses and suggested strategies for navigating such situations*.
6. A lack of control over IBD	Changing symptoms; Feeling unable to make plans; Need for contingency plan/flexibility; Fear of flare up; Controlling the controllable	North—Power of thoughts	*‘Parvi's story’ adapted to include scenarios such as worries about going to the toilet at work, feeling like a burden to friends and embarrassment around symptoms*.
		North— Managing uncertainty	*‘Ali's story’ edited to highlight uncertainty of daily symptoms, feeling a lack of control over your body and social withdrawal*.
		Across COMPASS programme	*Inclusion of IBD‐specific symptom examples throughout entire COMPASS programme, including in patient stories*.
		West—Managing stress	*Added guidance on setting boundaries and learning to say no in personal and professional relationships*.
7. Difficulty in disclosing or discussing IBD	Conversations with healthcare professionals; Talking to friends and family; Dealing with work; Dealing with unhelpful comments	South—Making use of professional support	*Luke's story’ edited to reflect IBD‐specific challenges, including feelings of embarrassment at medical appointments, and importance of having confidence to seek clarification about treatment and side effects*.
		West—I'm me not my LTC	*Edited ‘Self‐Kindness’ section to include content on dealing with reactions to IBD, focusing on avoiding internalising and including the ‘what would I say to a friend’ technique*.
8. Worries about the need for future surgery/procedures	Future surgery	New Resource— Preparing for procedure	*Extra worksheet resource includes tips and examples exploring anxiety and IBD, practical ways to prepare for procedures and distraction and grounding techniques for anxiety management*.
9. Understanding IBD and your body is important	Knowing what normal looks like; Understanding the link between IBD and mental health	West—Managing stress	*Additional content added on the gut‐brain axis overview, explaining the relationship between stress and gut symptoms*.
10. Social burden/disruption		West—I'm me not my LTC	*‘Sami's story’ edited to illustrate the impact of IBD symptoms, having to leave a family outing early because of symptoms and feelings of guilt around this*.
11. Sex and relationships	Intimacy; Dating and relationships; body image	South— Managing professional support	*‘Luke story’ edited to explore worries around dating, including how to disclose and discuss IBD symptoms with a new partner*.
12. Difficulty adjusting to diagnosis		Across COMPASS programme	*Acknowledgement of the challenge of coming to terms with a ‘new normal’ after diagnosis or a period of being well*.
13. IBD and mental health	Depression, Anxiety, Stress	Across COMPASS programme	*Focus on IBD‐specific negative thoughts and distress, adapting existing CBT techniques and content already to include IBD relevant examples where appropriate*.

### 3.3 Phase 3—Iterative Review Phase

The third phase involved further collaboration with the advisory group who reviewed the initial changes to the COMPASS‐IBD content. Across several rounds of feedback, the advisory group made 64 suggested changes, of which, 56 were actioned. Table 5 provides example comments from the advisory group and resulting adaptations (examples highlighted focus on suggested content changes rather than minor grammatical changes).

**Table 5:** Examples of Phase 3 advisory group feedback and resulting COMPASS‐IBD adaptations.

Example of feedback/suggestion from advisory group	Outcome based on feedback
Text needs to validate the difficulty of achieving personal goals when living with IBD.	Added a sentence acknowledging that achieving goals can be challenging when living with IBD.
Include examples of guilt during periods of poorer health and the impact this—e.g., ‘I should feel better than this’.	Added specific examples of worries/guild about lack of control, using suggested wording.
Add detail about the lack of understanding/judgement from people when needing to be near a toilet.	Specific example to address this added to ‘worries’ section.
More specificity around the nature of symptoms: ‘It's not always a choice to slow down, it's an important response that is necessary’.	Added text to clarify the importance of rest for IBD symptom management.
Patient story example was not specific enough to IBD challenges, focus more on symptoms such as incontinence and the impact this has on routine.	Patient story reworked, with additional context around the range of symptoms and the importance of creating ways to adjust to maintain quality of life.
Reword the section on stress and symptoms for clarity/to avoid invalidating people with functional symptoms.	Changed wording to clarify the link between stress and physical symptoms and how they interact.
Clarify discussion of ‘short term’ solutions. Legitimise and offer support for both shorter‐ and longer‐term planning.	Added sentence to explicitly talk about how both short‐ and long‐term mindset can be useful and helpful in different situations.
Acknowledgement needed that thinking/talking about emotions can be difficult (*the focus of the session)*. Should acknowledge this upfront where relevant and encourage users to take a break.	Added statements acknowledging that reflecting on feelings can be difficult and draining and encouraging users to take a break and reward themselves afterward.
Highlight the benefits of sharing thoughts and feelings with loved ones.	Added a statement about positive impact of disclosure in relationships and how this can strengthen understanding.
Highlight that loved ones might not always be aware/as knowledgeable about IBD and the impact of this.	Patient story edited to include an example of misunderstanding from family and how to navigate this.
Address how lack of regular, face‐to‐face contact with medical team can be challenging.	Patient story edited to include advocating for oneself in changing medical settings/team.
Feedback from dieticians that the food and diet section is a complex issue in IBD. A much larger/more specific piece of work or intervention would be required to fully address dietary and nutritional advice in IBD.	Changed approach to this section to focus on thoughts and feelings related to food and eating, rather than specific dietary advice. Content focused on how to manage situations that cause anxiety around food and eating.
Feedback from pharmacist suggested we should provide more specific advice for IBD medications.	Medication and side effect section expanded as per pharmacist recommendations.
Suggestion to explore examples of saying ‘no’ in the workplace to set boundaries to protect wellbeing	Added a specific worked example of setting boundaries at work regarding capacity to a patient story.

A table of all comments from the advisory group, alongside consequent changes to the programme, is provided in Supporting Information S1: Table S4. Advisory group feedback ranged from minor typographical changes (e.g., to clarify a point or change wording) to larger content changes covering a whole topic or approach to a section. For example, in the COMPASS section *West—Healthy Lifestyle*, dieticians in the advisory group advised that more specific dietary advice would be needed to provide sufficient nutritional information to support the variety of needs within the IBD population. As this was out of scope for the COMPASS intervention (which focuses on managing psychosocial wellbeing), this section was instead reworked to discuss attitudes toward eating and psychosocial aspects related to food, rather than providing any specific nutritional advice. This work highlighted the need for more specific, focused support around food and eating in IBD, leading the research team to develop a targeted intervention around food behaviours in IBD, which is currently underway.

Suggestions from the advisory group which were not actioned were those deemed out of scope for the COMPASS‐IBD intervention, such as those that did not align with CBT therapeutic goals or could not be accommodated within existing module formats. Broader examples were prioritised to ensure an inclusive range of IBD experiences. Healthcare professionals also provided advice on appropriate wording for clinical aspects, such as medication side effects, as well as on the formulation of content to support appropriate behaviour change within the clinical context. For example, in one module focused on quality of life and values, the psychologist highlighted how practical ‘safety behaviours,’ such as carrying a kit or identifying toilet locations, could enable social engagement and support valued activities. Once all changes were made, these were reviewed and finalised by the research team. Final document versions were sent to the web developer to be published on the new COMPASS‐IBD web platform for delivery ahead of the implementation study.

### 3.4 Phase 4—Post‐Process Reflection With PPI Members

Nine PPI members of the advisory group responded to our feedback survey (75% response rate). All members responded positively about their experience as a PPI member, with every respondent stating that they would take on a PPI role again and would not change anything about their involvement.

**Figure 2:**
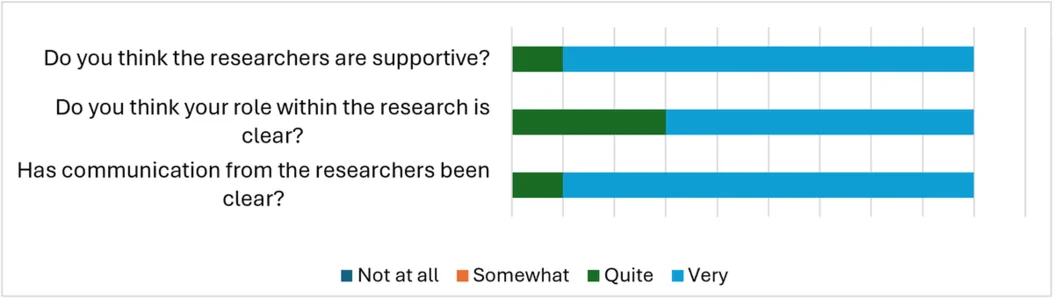
Feedback from PPI group on researcher support communication and role clarity.

Communication from the research team was viewed positively, with eight respondents rating it as *very clear* and one as *somewhat clear*. Clarity around their role within the research was also strong, with six respondents reporting it was *very clear*. All respondents indicated they had gained something valuable from their involvement, six to a *very great extent* and three to a *quite great extent* and nearly all respondents (*n* = 8) described the research team as *very supportive* (Figure 2).

**Figure 3:**
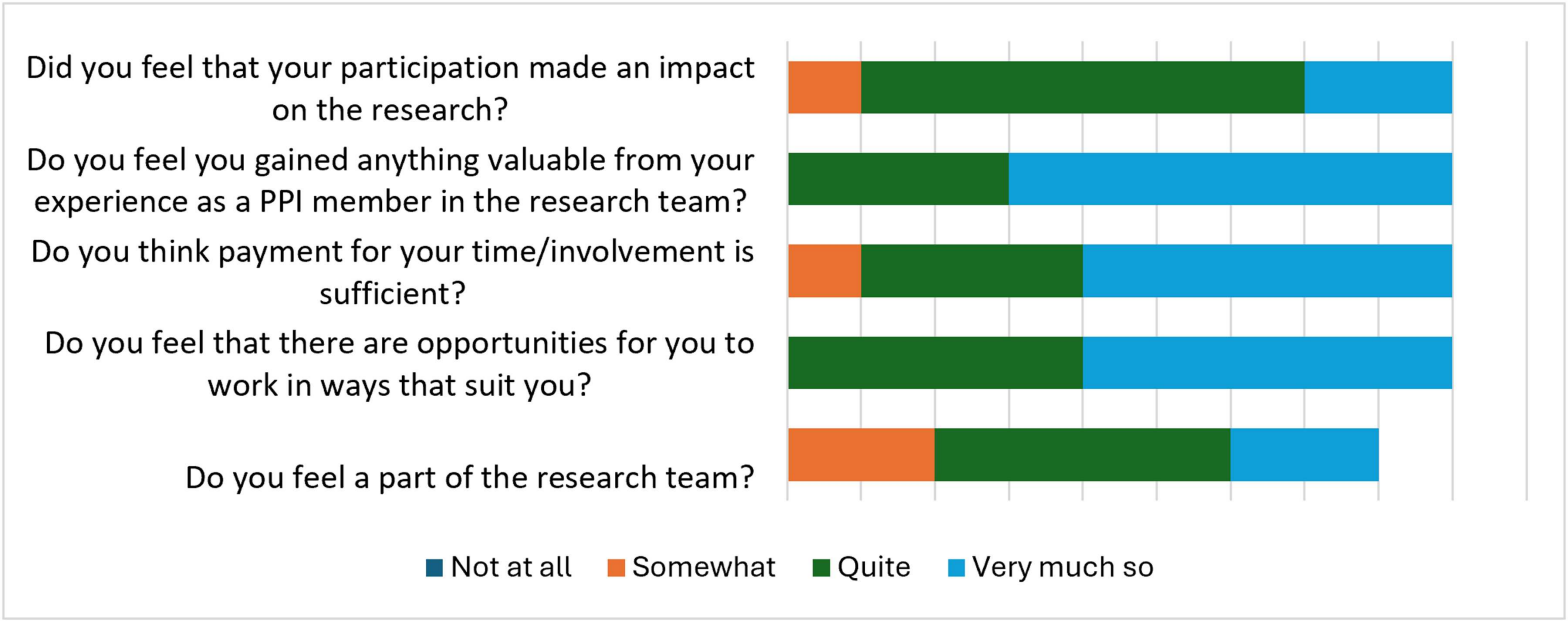
Feedback from PPI group members on their contribution, value and inclusion in the research team.

Perceptions of influence on the research were also positive but more varied as shown in Figure 3. Six respondents felt their participation had *somewhat* made an impact, two felt it had a *very clear* impact, and one reported *quite* an impact. Similarly, when asked about inclusion within the research team, four respondents reported feeling *quite* part of the team, two *very much*, and two *somewhat*.

In free‐text responses, PPI members emphasised the supportive nature of the research team, which enabled open and honest conversations.

I was surprised how supportive the researchers were… questions could be answered easily but also given enough space & empathy to feel I could answer freely.

They also valued being involved in decision‐making, which reinforced their sense of inclusion:

Being advised why things were being used instead of just dictated to certainly helped me feel a part of the research team.

The overall experience was described as positive and enriching, not only for contributing to research but also on a personal level for some.

The whole experience was a positive, enhancing and process, with the added real value of helping me to properly come to terms with my Crohn's disease.

When asked whether they disliked anything about their role as a PPI member, participants reported no negative experiences of working with the team. Some noted frustration at waiting for the outcome of the research, whilst another member reflected that conversations could occasionally lead to uncomfortable feelings and reflection on their own challenging experiences living with IBD, though these were understood as part of the process.

## 4 Discussion

This paper outlines a four‐step process for adapting the original COMPASS intervention into COMPASS‐IBD, a digital CBT‐based intervention targeting illness‐related distress tailored to pwIBD. Specifically, it details the collaboration with an advisory group (of PPI members with IBD and HCPs) throughout the adaptation process, to ensure relevancy and accessibility of content. Phase 1 identified thirteen overarching challenges of living with IBD, including the fear of having an accident, stigma and embarrassment around IBD, symptom burden, and worries around surgery or procedures. Phase 2 mapped these challenges to existing COMPASS content, to highlight potential content to be tailored, including adapting patient stories to experiences of living with IBD, utilising IBD‐specific worked examples of overcoming psychosocial challenges, and the development of new resources to support unique identified needs (e.g., managing unhelpful comments about IBD). The third phase involved iterative review of these adaptations with the advisory group, resulting in a further 64 suggested changes, of which 56 were incorporated. Substantive changes included revising the original COMPASS food and nutrition section away from generic nutritional advice towards a focus on thoughts and feelings related to food, as relevant to living with IBD. Phase 4 captured PPI members post‐adaptation reflections about their involvement in the process. Most members highlighted positive experiences including clear communication from the research team, supportive interactions, and feeling valued and included in decision‐making. Members perceptions of their influence in the research project however were generally positive but more varied.

In line with previous literature [29], incorporating those with lived experience in the development of COMPASS‐IBD provided rich insight into the psychosocial and practical challenges of living with IBD, which enhanced the relevance and authenticity of patient stories and examples. This was highlighted by our implementation study (reported separately [25]) that found that COMPASS‐IBD was acceptable and effective at improving anxious and depressive symptomology, with effect size improvements better than those previously observed in real‐world settings [22]. This may indicate that this collaborative, personalised approach resulted in a more relevant and acceptable intervention.

Several insights from our advisory group also highlighted areas for future research and support beyond the scope of the current COMPASS‐IBD intervention. For example, advisory group feedback on the impact of food and eating on social situations and relationships indicated the need for more targeted, specific support. HCPs similarly emphasised the importance of providing accurate, IBD‐specific nutritional guidance, highlighting food and diet as key areas for future development.

This study illustrates how collaboration with PPI members in intervention adaptation processes can be systematically captured and reported. Several studies describe meaningful PPI in intervention development for pwIBD [37, 38, 39] and there are examples of interventions that have been adapted for IBD [40], however most report high‐level intervention adaptations with little detail on how PPI influenced changes made [41]. Intervention adaptation provides a cost‐effective approach to tailoring pre‐existing interventions for new populations and is recommended by the MRC as a key consideration when developing interventions [31]. This paper therefore contributes to the literature by providing a detailed account of how PPI can play an important role in adapting a transdiagnostic intervention, demonstrating how lived‑experience and HCP insights can be identified, translated into intervention modifications and reviewed whilst preserving core therapeutic functions from the original intervention. Documenting these changes, following the key framework outlined by the MRC [31], can provide a transparent example of how PPI can inform intervention adaptation and provide a model for future digital intervention adaptations for other long‐term health conditions [42].

## 5 Limitations

Whilst the four‐phase process provided a pragmatic and person‐centred approach for documenting the COMPASS‐IBD adaptation, it was less prescriptive than established adaptation frameworks such as FRAME [42]. FRAME encourages detailed documentation of when, why and by whom adaptations are made, alongside consideration of their impact on intervention fidelity [42]. Our approach prioritised iterative collaboration with PPI and clinical partners and transparent reporting of how lived experience informed content changes, providing a detailed account of stakeholder influence on adaptation decisions informed by the PBA [26]. However, future studies may benefit from incorporating more formal adaptation frameworks alongside PPI approaches to capture adaptation decisions in greater detail.

Another important consideration when working with people with lived experience is ensuring diversity, so that a range of perspectives are included. Individuals from lower socioeconomic status groups are more likely to experience psychological comorbidities in LTCs [43] and the experience of healthcare can hugely vary based on race and ethnicity [44]. PPI representatives with lived experience should therefore include individuals from key target groups to ensure these voices are present in research and intervention development. Whilst specific efforts were made to recruit diverse PPI representatives in this study (including purposively targeting men with IBD and specific ethnic groups) the majority of PPI group members were white women (*n* = 8), with only two members from groups minoritized because of their ethnicity. Future work should consider additional approaches to ensure more diverse representation, which might include social media recruitment and outreach events to connect with priority groups. Although feedback from PPI was largely positive, it suggested that not all members felt fully clear about the impact of their contributions or consistently part of the research team. This highlights a need for future work to enact structured, transparent, and regular communication with patient partners, so they feel more integrated into the research process and understand how their role contributes to it.

## 6 Conclusion

This paper systematically outlines a four‐phase, person‐centred process for adapting a digital CBT intervention (COMPASS‐IBD) for people with IBD through structured collaboration with PPI and HCPs. It demonstrates how lived experience can meaningfully inform intervention adaptation and provides a transparent framework for embedding and documenting patient involvement in digital intervention development.

## Author Contributions

**Sophie Harding:** investigation, writing – original draft, methodology, writing – review and editing, data curation, project administration. **Harinder Singh:** funding acquisition, visualization, writing – review and editing. **Katrin Hulme:** conceptualization, investigation, funding acquisition, methodology, writing – review and editing, supervision. **Natasha Seaton:** investigation, methodology, writing – review and editing. **Abigail Wroe:** writing – review and editing, methodology. **Alexa Duff:** methodology, writing – review and editing. **Joanna Hudson:** conceptualization, investigation, funding acquisition, writing – review and editing, supervision. **Rona Moss‐Morris:** conceptualization, investigation, funding acquisition, writing – review and editing, visualization, supervision. **Annie S. K. Jones:** investigation, methodology, visualization, writing – review and editing, supervision, data curation.

## Ethics Statement

COMPASS has been developed under the control of the Kings Digital Therapies Quality Management System in compliance with the Medical Devices Directive 93/42/EEC for class I medical devices. COMPASS has all clinical safety and quality management protocols in place, including oversight by a digital clinical safety officer.

## Conflicts of Interest

R.M.M. receives grants from the MS Society, Crohn's and Colitis UK, and the NIHR. She is a beneficiary of a licence agreement between King's College London and Mahana Therapeutics and also receives consulting fees from Mahana Therapeutics. R.M.M. receives personal speaker fees from EAPM, BABCP, and Central and North West London NHS Foundation Trust. A.D. has received consulting fees/honoraria from Takeda, Alfa Sigma, Abbvie.

## Supporting information


Supporting File

## Data Availability

The data that support the findings of this study are available in the supporting information of this article.
