# Supplementary material for: Using a Person‐Centred Approach to Adapt a Digital Therapy for Inflammatory Bowel Disease (COMPASS‐IBD)
Source: Health Expect. 2026 Aug 21;29(4):e70818. doi: 10.1111/hex.70818 (PMC13495967; doi:10.1111/hex.70818)
Supplement: Supplementary file 1 [file HEX-29-e70818-s001.docx]

Supplementary Materials

Supplementary Table S1. GRIPP2-SF Checklist for Patient and Public Involvement (PPI)
Supplemental Table S2: Interview guide for Patient and Public Involvement (PPI) interviews

Supplemental Table S3: Patient and Public Involvement (PPI) Feedback survey items.
Supplemental Table S4: Examples of Phase 3 Patient and Public Involvement (PPI) feedback and resulting COMPASS-IBD adaptations.

Supplementary Table S1. GRIPP2-SF Checklist for Patient and Public involvement (PPI)

| Section and topic | Item |
| --- | --- |
| Aim | The aim of this study was to adapt an existing CBT-based intervention for illness related distress, into an illness specific version for people living with IBD, working with Patient and Public Involvement (PPI) advisors at each stage of the adaptation process. |
| Methods | This study had four phases, working with a PPI advisory group that included 12 people living with IBD and three healthcare professionals working clinically in IBD. The first stage involved a workshop and one-one discussions with PPI advisors, to develop a greater understanding of the patient experience and psychosocial challenges for IBD. These discussions informed changes made to the  IBD-specific version of the COMPASS programme and iteratively reviewed and discussed with PPI advisors for acceptability and suitability. A key member of the research team was a PPI collaborator, who supported the adaptation process throughout and the development of this manuscript. A final reflective phase aimed to capture the experiences of PPI advisors on the adaptation process, with an aim to understand |
| Study results | - PPI identified key issues of living with IBD, including the unpredictable nature of IBD, isolation, and the sigma and burden of symptoms. - Iteratively reviewed adaptations, providing feedback on wording changes and considerations of processes. 64 recommendations were made, with 54 actioned. - Provided video content of lived experience for the intervention - Provided reflections on the process of PPI, highlighting a positive experience. |
| Discussion and conclusions | PPI contributions shaped the adaptation process by informing inform key content, tone and the embedding of lived experience within patient stories and examples. Most of the suggested changes were implemented, highlighting the meaningful influence of PPI on the adaptations made. Gathering reflections from contributors further embedded them in the research process but highlighted some uncertainty about their role and the value of their input, indicating areas where communication could be improved in future collaborations. |
| Reflections and critical perspective | Collaboration with PPI worked well and had a significant impact on the adaptation process. The flexible, iterative approach to feedback and consultation allowed for meaningful feedback at each stage of adaptation. Implementing the changes and communicating decisions to PPI members helped to build trust and a sense of impact. However, reflective feedback highlighted that researcher communication around the role and value of PPI could have been more explicitly shared, as some contributors were less clear about how their role helped to shape the work. Despite efforts, there was also a lack of diversity in the advisory group, limiting the range of perspectives. In future work, we need to prioritise more inclusive recruitment strategies, including outreach within the community to ensure that under‑represented groups have equitable opportunities to be involved. |

Supplemental Table S2: Interview guide for Patient and Public Involvement (PPI) interviews

| Introduction | |
| --- | --- |
| Introductions and thanks  Brief overview of Compass  reason for interview (adapting Compass programme to people living with IBD) | |
| Questions | Prompt |
| Living with IBD   - Could you tell me a little bit about your IBD and the journey you’ve been on with it? | - Could you tell me what you feel are the main challenges you face living with IBD? |
| The first section of COMPASS focuses on change and uncertainty.  Can you tell me a bit about how you’ve had to deal with change or unpredictability as part of your IBD? | |
| Our second section is focused around how you’re able to manage your day-day routine and symptoms.  How does IBD impact your day-day life? | |
| What are the main symptoms, if any, you manage? | - How do these impact your daily routine? - How do they affect your mood and emotions? - How do you go about managing your symptoms? |
| How do you go about managing your symptoms? | - Do you have any tips or tricks that you find help you to manage your symptoms? |
| Are there any emotions that come to mind when you think about your condition? | - Has this changed over time? - How so? |
| The third section of compass focuses on relationships and support.   Do you receive any support for your condition? | - Professional? - Personal? - Has your IBD affected your relationships? |
| - How do your (family/friends/partner) respond to your condition? | - What do they do well - How could they improve? |
| - (if relevant) How do work respond to your condition? | - Do you feel supported? - How do they support you? - What do they do well/how could they improve? |
| - How are how are interactions with your IBD team/healthcare professionals? | - Do you feel supported? - How do they support you? - What do they do well/how could they improve? |
| Finally, the last section of compass focuses on how your condition affects other parts of your lifestyle like diet, sleep and exercise.  Can you tell me about your IBD and how it impacts on your:  Sleep  Diet  Exercise  Medication management | |
| Do you have any tips or strategies help you manage your condition? | Tips for employment  Social life  Relationships  Holidays |
| Closing:  Is there anything you would like to add?  Closing remarks and thank you | |

Supplemental Table S3: Patient and Public Involvement (PPI) Feedback survey items.

| Survey items | Response format |
| --- | --- |
| What made you interested in getting involved in research as a PPI member? | Free text response box |
| Has communication from the researchers been clear? | Scale: Not at all, Somewhat, Quite, Very |
| *If you have anything you would like to add about communication with the team, please add here* | Free text response box |
| Do you think your role within the research is clear? | Scale: Not at all, Somewhat, Quite, Very |
| *If you have anything you would like to add about how clear your role in the research was, please add here:* | Free text response box |
| Do you think the researchers are supportive? | Scale: Not at all, Somewhat, Quite, Very |
| *If you have anything you would like to add anything about support from the researchers, please add here:* | Free text response box |
| Do you feel a part of the research team? | Scale: Not at all, Somewhat, Quite, Very |
| *If you would like to add anything about feeling part of the research team, please add here. Learning more about what made you feel included/not included is really helpful to the team to ensure we can keep developing valuable partnerships.* | Free text response box |
| Do you feel that there are opportunities for you to work in ways that suit you? | Scale: Not at all, Somewhat, Quite, Very |
| *If you would like to add anything about the work, either about the workload or the way in which you got involved with research, please add here:* | Free text response box |
| Do you think payment for your time/involvement is sufficient? | Scale: Not at all, Somewhat, Quite, Very |
| *If you would like to add anything about payment, please add here:* | Free text response box |
| Do you feel you gained anything valuable from your experience as a PPI member in the research team? | Scale: Not at all, Somewhat, Quite, Very |
| *If you would like to add anything about how valuable (or not valuable) you found the experience, please add here:* | Free text response box |
| Did you feel that your participation made an impact on the research? | Scale: Not at all, Somewhat, Quite, Very |
| *If you would like to add anything about how you felt about your impact on the research, please add here:* | Free text response box |
| Do you dislike anything about being a PPI member? If so, what do you dislike? | Free text response box |
| Do you like anything about being a PPI member? If so, what do you like? | Free text response box |
| Would you change anything about your experience working as a PPI member? | Yes, No, Maybe |
| *Please could you explain why? (For any response)* | Free text response |
| Would you work as a PPI member in future research? | Yes, No, Maybe |

Supplemental Table S4: Examples of Phase 3 Patient and Public Involvement (PPI) feedback and resulting COMPASS-IBD adaptations.

| **Example of feedback/suggestion from advisory group** | **Outcome based on feedback** |
| --- | --- |
| 1. Text needs to validate the difficulty of achieving personal goals when living with IBD | 1. Added sentence to acknowledge that goals can be difficult to achieve when living with IBD. |
| 2. Include examples of guilt around being unwell and the impact this has when going through a period of poorer health – e.g. ‘I should feel better than this’ | 2. Added specific examples of worries. Used wording suggested in comment to highlight guilt about not being in control of IBD. |
| 3. Add detail about the lack of understanding/judgement from people when needing to be near a toilet | 3. Specific example to address this added to ‘worries’ section |
| 4. More specificity around the nature of fatigue and symptoms - ‘It’s not always a choice to slow down, it’s an important response that is necessary’ | 4. Added text to clarify the importance of rest for IBD symptom management. |
| 5. Patient story example was not specific enough to IBD challenges. Suggested to focus more on symptoms such as incontinence and the impact this has on routine. | 5. Patient story reworked, including providing additional context given before the example to explain the range of IBD symptoms and the importance of creating ways to adjust to maintain quality of life. |
| 6. Reword the section discussing stress and symptoms to improve clarity and avoid invalidation of people living with functional symptoms. | 6. Changed wording to clarify the link between stress and physical symptoms and how they interact. |
| 7. Clarify discussion of ‘short term’ solutions. Legitimise and offer support for both shorter- and longer-term planning. | 7. Added sentence to explicitly talk about how both short- and long-term mindset can be useful and helpful in different situations. |
| 8. Acknowledgement needed that thinking/talking about emotions can be difficult (*the focus of the session).* Should acknowledge this upfront where relevant and encourage users to take a break if needed. | 8. Added in statements at the start, midway and end of sessions to validate that thinking about feelings can be difficult and draining. Encouraged users to take a break and reward themselves for completing this section. |
| 9. Highlight the benefits of sharing thoughts and feelings with loved ones | 9. Added in a statement about the dual impact of disclosure on relationships and how this can strengthen understanding. Also emphasised this in relevant patient story. |
| 10. Highlight that loved ones might not always being aware/as knowledgeable about IBD and the impact of this. | 10. Patient story edited to include an example of misunderstanding from the family and how to navigate this. |
| 11. Address how lack of regular, face-to-face contact with medical team can be challenging. | 11. Patient story edited to include advocating for self in changing medical settings/team |
| 12. Feedback from dieticians that the food and diet section is a complex issue in IBD. A much larger/more specific piece of work or intervention would be required to fully address dietary and nutritional advice in IBD. | 12. Changed approach to this section to focus on thoughts and feelings related to food and eating, rather than specific dietary advice. Content focused on how to manage situations that cause anxiety around food and eating. |
| 13. Feedback from pharmacist suggested we should provide more specific advice for IBD medications. | 13. Medication and side effect section expanded as per pharmacist recommendations |
| 14. Suggestion to explore examples of saying “no” in the workplace to set boundaries to protect wellbeing | 14. Added a specific worked example of setting boundaries at work regarding capacity to a patient story. |
